# Influence of 2′-fucosyllactose and galacto-oligosaccharides on the growth and adhesion of *Streptococcus mutans*

**DOI:** 10.1017/S0007114520001956

**Published:** 2020-10-28

**Authors:** K. Salli, E. Söderling, J. Hirvonen, U. K. Gürsoy, A. C. Ouwehand

**Affiliations:** 1 DuPont Nutrition & Biosciences, Kantvik, Finland; 2 Faculty of Medicine, Institute of Dentistry, University of Turku, Turku, Finland

**Keywords:** 2′-Fucosyllactose, Galacto-oligosaccharides, *Streptococcus mutans*, Human milk oligosaccharides, Xylitol, Adhesion

## Abstract

Human milk oligosaccharides, such as 2′-fucosyllactose (2′-FL), and galacto-oligosaccharides (GOS), a prebiotic carbohydrate mixture, are being increasingly added to infant formulas, necessitating the understanding of their impact on the oral microbiota. Here, for the first time, the effects of 2′-FL and GOS on the planktonic growth and adhesion characteristics of the caries-associated oral pathogen *Streptococcus mutans* were assessed, and the results were compared against the effects of xylitol, lactose and glucose. There were differences in *S. mutans* growth between 2′-FL and GOS. None of the three *S. mutans* strains grew with 2′-FL, while they all grew with GOS as well as lactose and glucose. Xylitol inhibited *S. mutans* growth. The adhesion of *S. mutans* CI 2366 to saliva-coated hydroxyapatite was reduced by 2′-FL and GOS. Exopolysaccharide-mediated adhesion of *S. mutans* DSM 20523 to a glass surface was decreased with 2′-FL, GOS and lactose, and the adhesion of strain CI 2366 strain was reduced only by GOS. Unlike GOS, 2′-FL did not support the growth of any *S. mutans* strain. Neither 2′-FL nor GOS enhanced the adhesive properties of the *S. mutans* strains, but they inhibited some of the tested strains. Thus, the cariogenic tendency may vary between infant formulas containing different types of oligosaccharides.

Human milk oligosaccharides (HMOs), a diverse group of carbohydrates, are the third most abundant component in human milk^(1)^. More than 150 HMO structures have been identified in human breast milk, of which 2′-fucosyllactose (2′-FL; trisaccharide of fucose, galactose and glucose) is the most abundant HMO^(1,2)^. The concentration of 2′-FL in human milk is approximately 2·7 g/l, although it can vary, depending on the time of lactation^(2)^. Only a small proportion of HMOs are absorbed, and most are fermented in the colon by the microbiota, primarily by specific bifidobacteria, such as *Bifidobacterium longum* subsp. *infantis* and *Bifidobacterium bifidum* ^(1)^. HMOs function as selective prebiotics, improve immune development in the host and inhibit the adhesion of certain pathogens in the gut^(1,3,4)^. Recent advances in the production of HMOs have allowed 2′-FL to be included in infant formulas to better approximate the composition of breast milk^(5,6)^, since fucosylated oligosaccharides are nearly absent from bovine milk which is used as basis for infant formulas^(7)^. Galacto-oligosaccharides (GOS) are a mixture of lactose-based oligosaccharides with varying lengths and linkages between glucose and galactose, the terminal unit being glucose^(8)^. GOS are often used in infant formulas to mimic the prebiotic features of human milk^(8–10)^.

Dental caries is a common multifactorial disease, in which bacteria in a biofilm produce acids that dissolve enamel^(11)^. *Streptococcus mutans* is one of the most extensively studied cariogenic bacteria. *S. mutans* is well adapted to thrive under biofilm conditions that alter bacterial metabolism and decrease the susceptibility to host defence and antimicrobials^(11–13)^. *S. mutans* produces acids efficiently, creates a low-pH environment, utilises various carbon sources and can adhere to the tooth surface through the production of extracellular glucans and adhesins^(11,14)^. The acquisition of mutans streptococci (i.e. *S. mutans* and *Streptococcus sobrinus*) at an early age is a risk factor for caries development later in life, the risk of which can be decreased through caries prevention strategies early in the prenatal and postnatal phases^(15,16)^. Several mechanisms have been proposed to interfere with the virulence of *S. mutans*, such as inhibiting adhesion, preventing growth through non-fermentable carbon sources and affecting biofilm formation^(17,18)^. There are limited data on the effects of HMOs and GOS on *S. mutans* and other oral bacteria. However, GOS are structurally different from the oligosaccharides naturally occurring in human milk^(19)^.

Breast milk components can alter the composition of the oral microbiota, several of which have been examined with regard to their effects on growth, adhesion and biofilm formation in *S. mutans* ^(20–22)^. Infant formulas contain caries-protective components, such as proteins and Ca, but the carbohydrates in them may increase the risk for caries, especially if bottle feeding is taking place *ad libitum*, several times a day.

Because GOS and, increasingly, HMOs are added to infant formulas, there is an urgent need to determine the influence of these carbon sources on cariogenic bacteria and how they affect the bacterial colonisation on teeth. We hypothesised that mutans streptococci can use HMOs and GOS as sources of carbohydrate to increase their growth and adhesion. Thus, the aim of the present study was to examine whether a specific HMO, 2′-FL and GOS – in the form of a commercial prebiotic – shape the growth and adhesion of three strains of the dental pathogen *S. mutans in vitro* and compare the results to those of xylitol and lactose.

## Materials and methods

### Micro-organisms

Three strains of *S. mutans* were tested: the type strain DSM 20523 (ATCC 25175), Ingbritt and the clinical isolate CI 2366. The origin, isolation and identification of the clinical isolate have been described earlier^(23,24)^.

### Test substrates

A 10 % (w/v) suspension of 2′-FL (DuPont Nutrition & Biosciences and Inbiose), xylitol (DuPont Nutrition & Biosciences), glucose (J. T. Baker), lactose (Sigma-Aldrich) and GOS (kindly provided by Clasado Biosciences) were prepared in sterile water, sterile-filtered (0·2 μm Minisart; Sartorius AG) and stored at −20°C until use.

### Growth experiments

Bacterial growth experiments were performed as previously described^(25)^. Briefly, *S. mutans* strains were revived from frozen (−70°C) stocks and subcultured in glucose-containing Brain Heart Infusion medium (BHI) (LAB049; LabM Ltd) or Tryptic Soy Broth (TSB) (Bacto™; Becton Dickinson and Company) at 37°C overnight. After the subculture, bacteria were inoculated in fresh TSB growth medium with glucose and incubated at 37°C overnight. Cell suspensions (1 % (v/v)) were prepared in modified TSB without carbohydrates and used immediately for the growth assays.

2′-FL, xylitol, GOS, lactose or glucose solution (10 %) were added to each well of the plate (20 µl), and the wells were filled with suspensions of a single bacterial strain (180 µl). Thus, the final concentration of the carbohydrate substrate in each well was 1 % (w/v). Modified TSB without any added carbohydrates (sterile water was added instead of test substances) was used as a control. Bacterial growth was monitored by measuring the optical density at 600 nm every 30 min for 24 h on a Bioscreen^©^ C system (Labsystems) in an anaerobic cabinet (80 % N_2_, 10 % CO_2_, 10 % H_2_). The area under the growth curve over 24 h was used to quantify growth^(25)^. Three independent experiments, each performed in triplicate, were run.

### Adhesion to hydroxyapatite

Adhesion to hydroxyapatite (HA) was evaluated per Haukioja *et al.* ^(26)^. Parotid saliva was collected on ice each morning before the experiments using Lashley cups by stimulation with a Salivin lozenge (Pharmacia Ltd). Parotid saliva was diluted 1:1 with buffered KCl (50 mm KCl, 0·35 mm K_2_HPO_4_, 0·65 mm KH_2_PO_4_, 1·0 mm CaCl_2_, 0·1 mm MgCl_2_ and pH 6·5) before the experiments. The diluted saliva was used to coat the HA powder (Clarkson Chromatography Products Inc.), after which the HA was washed three times before bacteria were added. *S. mutans* was first grown overnight in BHI (Becton Dickinson) at 37°C and then for 3–4 h until the mid-logarithmic phase with 5 µl (50 µCi) ^35^S-labelled methionine (Perkin Elmer LifeSciences, Inc.). After growth, the bacteria were washed three times and suspended in phosphate-buffered KCl to approximately 10^8^ colony-forming units/ml. Suspensions (125 µl) of labelled bacteria and 1 % (w/v) 2′-FL, xylitol, GOS, lactose and plain buffer as a control were added to HA, to which the bacteria were allowed to adhere for 60 min under gentle agitation (IKA loopster; IKA®-Werke GmbH & Co. KG). Unbound bacteria were removed through three washes with 125 µl buffer. Labelled bacteria that adhered to HA were determined on a scintillation counter (MicroBeta 1450; Perkin Elmer Wallac) using scintillation cocktail (Optiphase Supermix/Optiphase Hisafe 3; PerkinElmer). Experiments were performed in six replicate wells (of which the lowest and highest values of scintillation counts were omitted to decrease variability; four replicates per individual experiment were used) and repeated at least twice. To combine experiments, the controls were set to 1, and the relative changes from the control were calculated.

### Adhesion to glass surface

Exopolysaccharide-mediated adhesion of *S. mutans* strains to a smooth glass surface was examined per Mattos-Graner *et al.* ^(27)^. *S. mutans* was first grown in BHI (LabM Ltd) at 37°C overnight. Then, triplicate samples were cultured in BHI with 1 % (w/v) sucrose (Suomen Sokeri Oy) and 1 % (w/v) 2′-FL, xylitol, GOS, lactose or plain buffer in a glass tube at a 30° angle in an anaerobic atmosphere at 37°C for 18 h. The control sample was comprised of BHI with sucrose. After incubation, the tubes were mixed gently, and unbound bacteria were transferred to another tube (planktonic bacteria). The glass tubes were rinsed with potassium phosphate buffer (0·05 mol/l, pH = 7) and remixed, and unbound bacteria were again transferred to the tube with planktonic bacteria. Then, potassium phosphate buffer was added to the glass tubes to quantify the bacteria that adhered to the glass. The planktonic bacteria tubes were centrifuged 6500 rpm for 5 min (Heraeus Biofuge Stratos, Kendro Laboratory Products), and the pellets were resuspended in potassium phosphate buffer. All tubes were vortexed and sonicated for 30 s (Q Sonica Sonicator LCC), and OD_550_ (optical density at wavelength of 550 nm) values were measured against potassium phosphate buffer on an Ensight plate reader (Perkin Elmer). The percentage of adherent bacteria was calculated as the ratio of adhered bacteria to all bacteria. The data are the composite of three independent experiments. In each experiment, the treatments were performed in triplicate. To combine and compare the experiments, the controls were set to 1, and the relative changes from the control were determined.

### Statistics

In growth experiments, statistical differences between treatment groups were analysed by one-way ANOVA and Dunnett’s multiple comparison test. In adhesion experiments, statistical differences between treatment groups were analysed by one-way ANOVA and Tukey’s multiple comparison test. The statistical analysis was performed using GraphPadPrism version 8.1 for Windows (GraphPad Software). *P* values of <0·05 were considered to be significant.

## Results

**Fig. 1.**
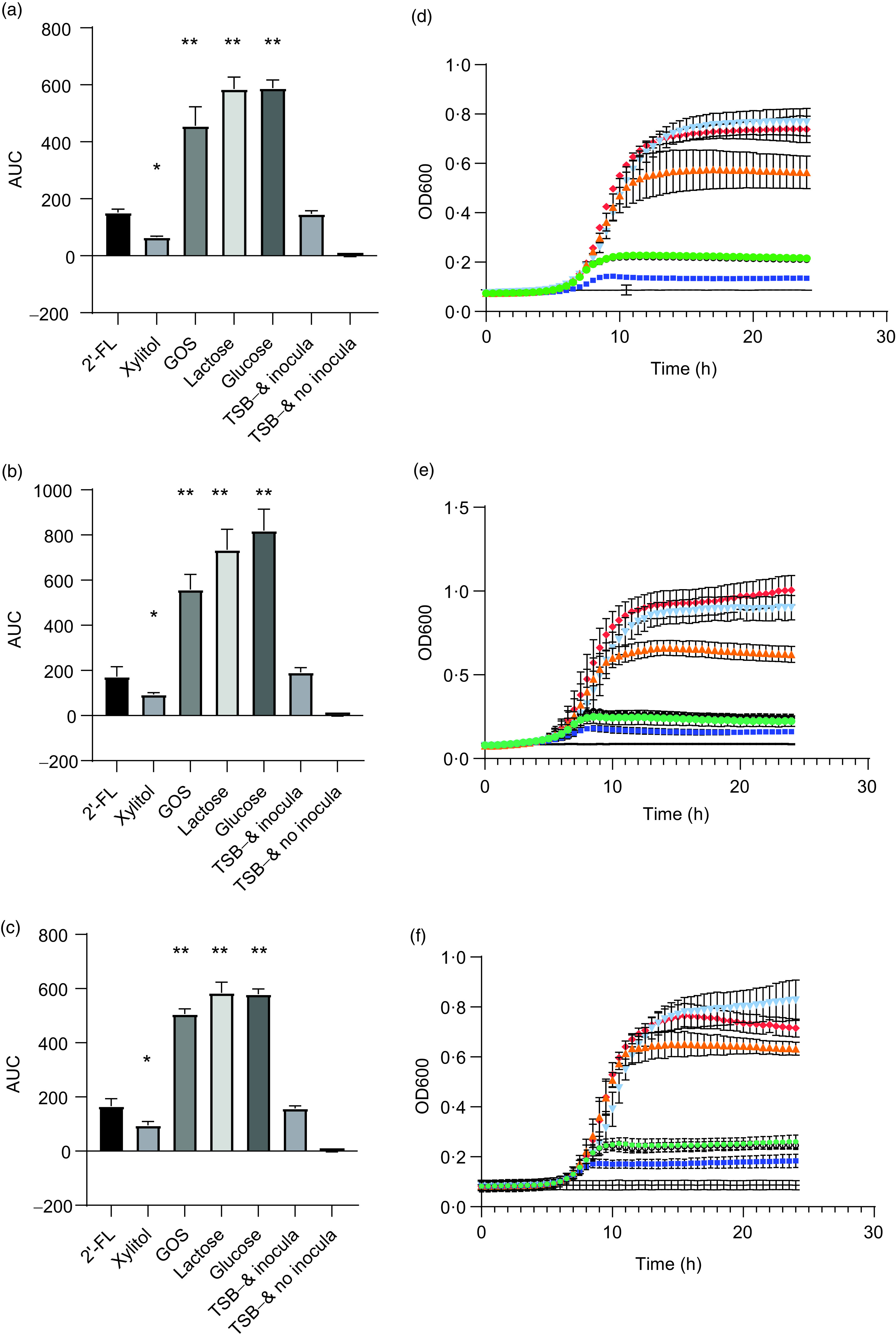
Area under the growth curve (AUC) (a–c) and growth curves (e and f) of *Streptococcus mutans* strains DSM 20523 (a and d), CI 2366 (b and e) and Ingbritt (c and f). Growth media contained 1 % (w/v) 2′-fucosyllactose (2′-FL), xylitol, galacto-oligosaccharides (GOS), lactose or glucose added to modified Tryptic Soy Broth (TSB−) devoid of glucose or other carbon sources. Values are means and standard deviations from three individual experiments, each with three replicates. * *P* < 0·05; ** *P* < 0·001 compared with TSB− and inocula. OD, optical density. 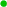, 2′-FL; 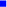, xylitol; 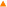, GOS; 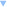, lactose; 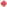, glucose; 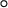, TSB− and inocula; 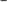, TSB− and no inocula.

*S. mutans* strains DSM 20523, CI 2366 and Ingbritt were unable to grow with 2′-FL as the sole carbon source, comparable with the result when no carbon source was added to the TSB growth medium (*P* > 0·05 for all strains, Fig. 1). In contrast, all *S. mutans* strains grew with 1 % GOS, 1 % lactose or 1 % glucose as a carbon source (*P* < 0·001, for all strains). Xylitol inhibited the growth of all three *S. mutans* strains *v.* the control without any added carbohydrates (*P* < 0·05, Fig. 1).

**Fig. 2.**
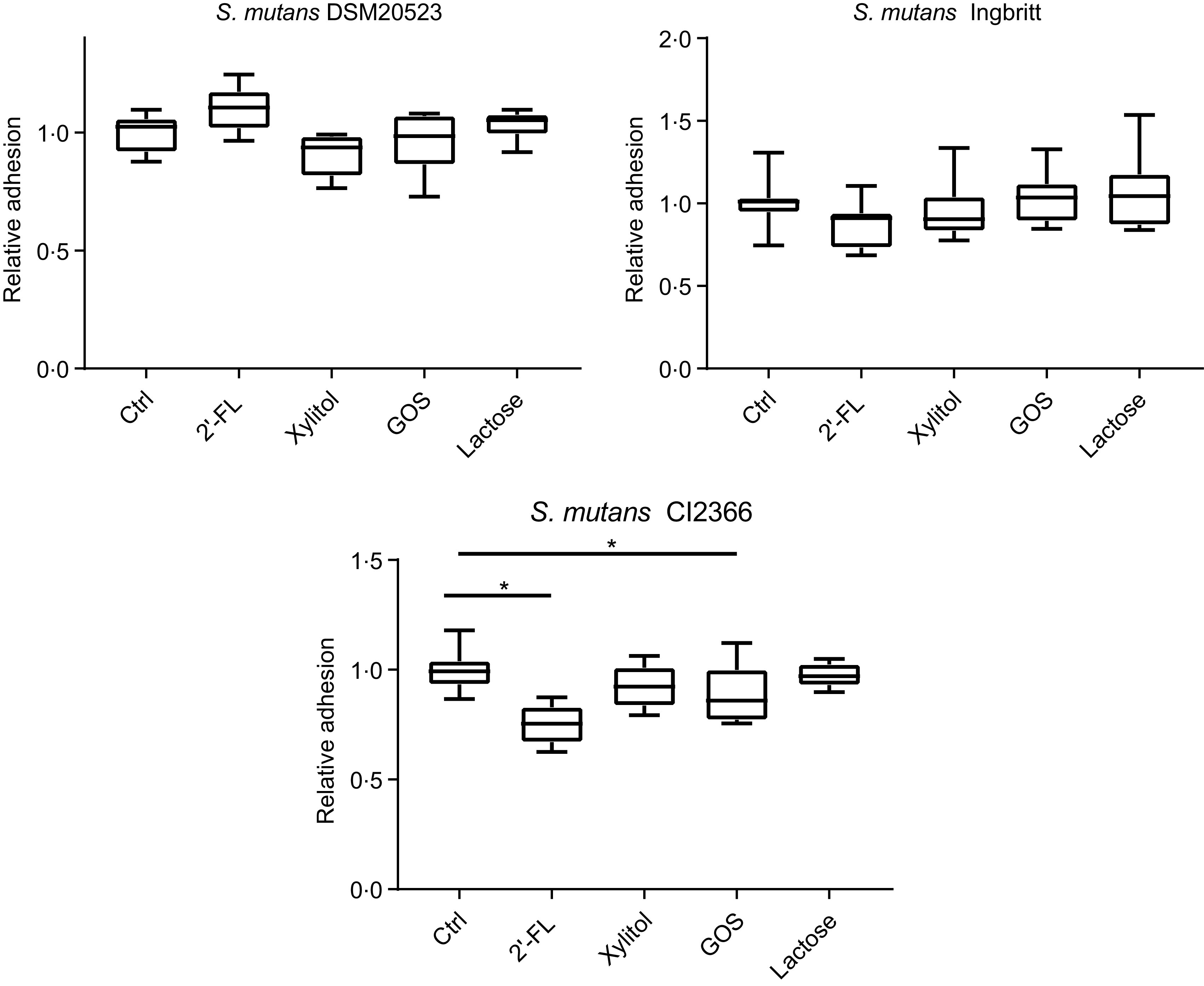
Relative adhesion to parotid saliva-coated hydroxyapatite (HA) for three *Streptococcus mutans* strains (DSM 20523, Ingbritt and CI 2366) with 1 % (w/v) 2′-fucosyllactose (2′-FL), xylitol, galacto-oligosaccharides (GOS), lactose and phosphate buffer as a control (Ctrl). Bacterial adhesion to HA was determined by scintillation count. *P < 0·05 compared with control.

The effects of 2′-FL, xylitol, GOS and lactose on *S. mutans* adhesion to parotid saliva-coated HA were evaluated (Fig. 2). There were no significant changes in strains DSM 20523 or Ingbritt *v.* the control with any of the carbohydrates (*P* > 0·05). Nevertheless, the adhesion of strain CI 2366 decreased significantly with 2′-FL and GOS compared with the control (*P* < 0·001 and *P* = 0·04, respectively).

**Fig. 3.**
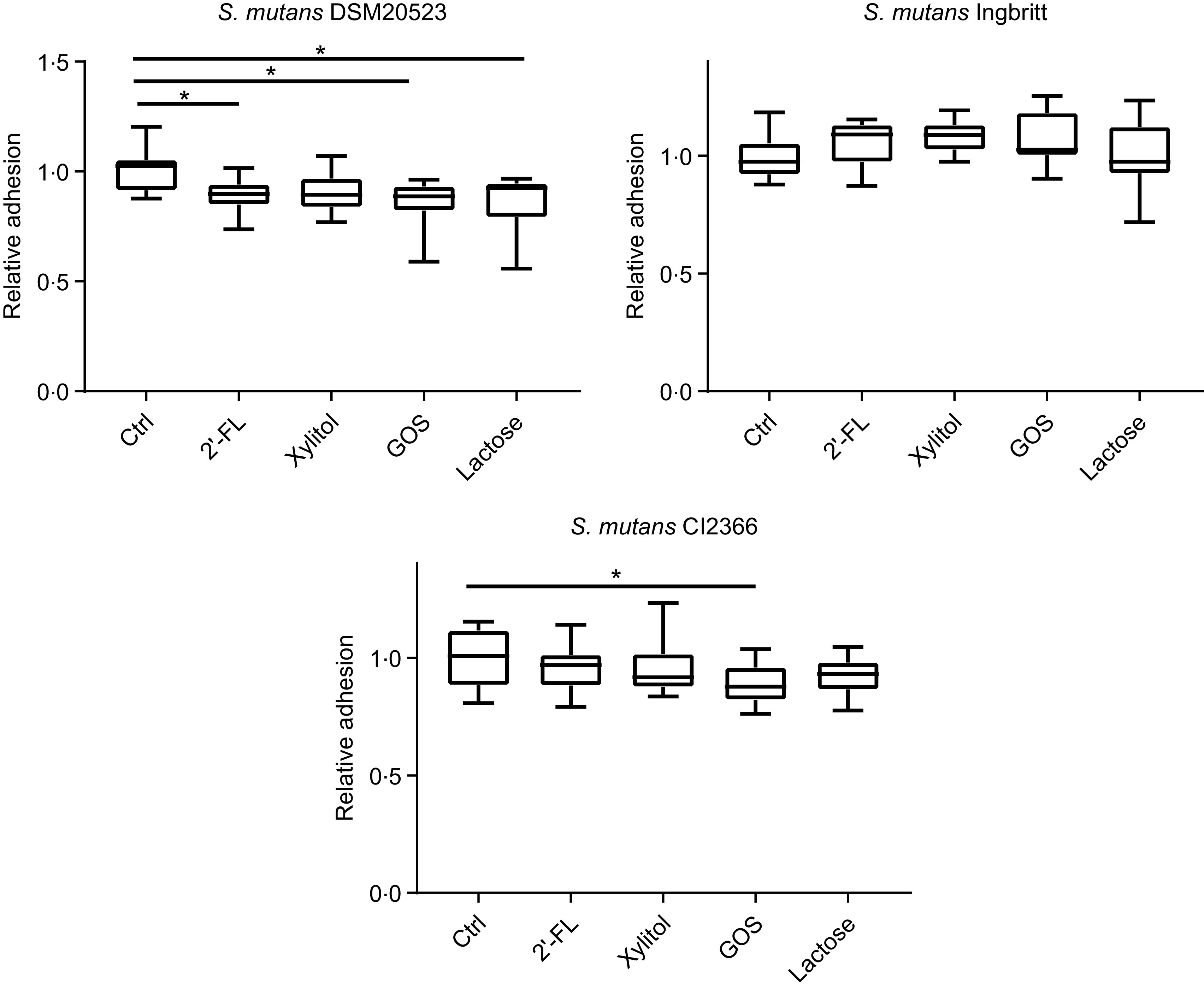
Relative adhesion of three *Streptococcus mutans* strains (DSM 20523, Ingbritt and CI 2366) to a glass surface. *S. mutans* strains were cultured in Brain Heart Infusion medium with 1 % (w/v) sucrose and 1 % (w/v) 2′-fucosyllactose (2′-FL), xylitol, galacto-oligosaccharides (GOS), lactose and buffer as a control (Ctrl). *P < 0·05 compared with control.

The effects of 2′-FL, xylitol, GOS and lactose on *S. mutans* adhesion to a glass surface – indicative of hydrophilic binding through exopolysaccharides – were also evaluated. The adhesion of strain DSM 20523 was reduced by 2′-FL (*P* = 0·02), lactose (*P* < 0·001) and GOS (*P* < 0·001) *v.* the control (Fig. 3). The adhesion of strain Ingbritt to glass was unaffected by any of the carbohydrates (*P* > 0·05), whereas that of strain CI 2366 decreased only with GOS (*P* = 0·02) (Fig. 3).

## Discussion

Human breast milk contains caries-protective factors^(28–30)^, but the effects of individual HMOs on oral health-related variables have not been reported. Our study is the first report on the effects of an individual HMO on *S. mutans*, an oral bacterium associated with the pathogenesis of caries. None of the three *S. mutans* strains that we tested utilised 2′-FL as a carbon source, whereas they grew well with GOS, lactose and glucose. *S. mutans* is well adapted to thrive in the oral cavity and can metabolise many carbon sources^(11,23,31,32)^. Thus, we aimed to determine whether *S. mutans* grows on HMOs and other prebiotics, such as GOS, which are also added to infant formulas. 2′-FL has been studied in infant formula at 0·2–1 g/l, for other health indications than oral health, as have GOS levels at 0·2–0·5 g/dl^(5,6,9,10,33)^. The 2′-FL concentrations that we tested exceeded those in infant formula and were slightly higher than GOS concentrations.

Our results are consistent with earlier studies showing that *S. mutans* utilise galactose, lactose and glucose^(31,32)^. Although limited data exist regarding GOS and cariogenic bacteria, *S. mutans* strain MT8148R (serotype c) was found earlier to ferment GOS similarly to our strains in the present study^(34)^. As reported at low xylitol concentrations, xylitol inhibited the growth of planktonic *S. mutans* strains^(23)^. Thus, while *S. mutans* can grow on glucose and galactose, the building blocks of both lactose and GOS, it failed to grow with the trisaccharide 2′-FL. The structure of 2′-FL contains fucose attached to lactose with α-1,2 linkage^(1)^. GOS are a mixture of varying length galactose units with lactose at the reducing end, with glycosyl linkages commonly either β-1,3, β-1,4 or β-1,6 depending on the enzyme used^(35)^. The growth of oral pathogens has not earlier been studied with HMOs, but potentially pathogenic Enterobacteriaceae and *Clostridium perfringens* did not grow using 2′-FL^(36,37)^. In addition, pooled HMOs have been shown to inhibit the growth of *Streptococcus agalactiae* CNCTC 10/18 (group B *Streptococcus*)^(38)^. However, the inhibition rate varied depending on HMO donors (and thereby the HMO composition). Degradation of HMOs requires an extensive set of glycoside hydrolases, membrane transporters and carbohydrate binding proteins^(39)^. Absence of these key proteins has been shown to result in the lack of ability to grow on HMOs. Bacteria, such as bifidobacteria or *Bacteroides*, which are able to grow with HMOs like 2′-FL, mainly apply one of the two strategies. *B. longum* subsp. *infantis* has several glycoside hydrolases, ATP binding cassette (ABC) transporters and extracellular solute binding proteins, it utilises to import HMOs into the cell and degrade oligosaccharides intracellularly^(39–41)^. *Bacteroides*, on the other hand, have been proposed to degrade complex HMOs partly and then transport processed oligosaccharides inside the cell for further degradation^(39)^. The α-1,2-fucosidase is required for utilising 2′-FL. However, its presence does not always show as utilisation of 2′-FL^(42)^. Recently, *Streptococcus pneumoniae* has been shown to contain two α-fucosidases^(43)^. The genome sequence of *S. mutans* strain UA159 is known to have four ABC transporters^(44)^, but transcription profiles for fucose or HMOs were not studied. Our results indicate that the studied *S. mutans* strains do not express the enzymes or other factors required to metabolise α-1,2 fucosylated carbohydrates. Consequently, 2′-FL should have no negative impact on the cariogenic potential of infant formulas, whereas other less selective prebiotic oligosaccharides may have.

The two adhesion experiments showed no consistent patterns of inhibition for 2′-FL or GOS. Notably, the adhesion of the virulent clinical isolate *S. mutans* Cl 2366^(24)^ to saliva-coated HA was reduced by 2′-FL and GOS. Further, exopolysaccharide-mediated adhesion to the glass surface decreased for *S. mutans* DSM 20523 with 2′-FL and GOS. HMOs have been suggested to function in the gut as selective prebiotics, antimicrobials, to inhibit the adhesion on colonic epithelium of some pathogens, for example, *Escherichia coli*, *Campylobacter jejuni* or protozoan parasite like *Entamoeba histolytica* and to modify host responses^(1,45)^. However, their effects are structure- and bacterium-specific^(3,4,45)^. HMOs have structural similarities to glycan structures in the gut epithelium, and they can function as decoy receptors, hindering the binding of pathogens^(4,45)^. To our knowledge, the effects of HMOs on adhesion to oral bacteria have not been studied before. The binding of *S. mutans* with substrates in salivary pellicle is mediated by sucrose-independent, adhesin-receptor interactions^(11,26)^. The adhesion of the two reference strains to saliva-coated HA was not significantly affected by 2′-FL or GOS, but both compounds significantly decreased the adhesion of strain CI 2366^(24)^. Earlier, other components of human milk casein, IgA and IgG have been shown to decrease the adhesion of *S. mutans* to saliva-coated HA^(21,22)^. Also, in earlier studies, changes in adhesion have been observed between *S. mutans* strains^(21)^.

Adhesion to a smooth glass surface, reflecting sucrose-dependent, extracellular glycan production-mediated adherence^(11,27,34)^, decreased or was unchanged with 2′-FL and GOS. These results are consistent with those from a similar experiment, showing that the adherence of *S. sobrinus* 6715 and *S. mutans* MT8148R was impeded by GOS^(34)^. However, for 2′-FL, our work is the first report to document the effects on exopolysaccharide-mediated adhesion in *S. mutans*. The two methods used in the present study evaluated two different *S. mutans* virulence factors. However, because of the strain-dependent variation in the results, further research is needed to clarify the effects of 2′-FL and other HMOs on *S. mutans* and other oral bacteria.

Xylitol did not affect the adhesion of *S. mutans* in any experimental setting, although its chemical properties may have suggested so. Xylitol binds water molecules and Ca ions, among other compounds, but is inert in other aspects^(46)^. The effects of xylitol on adhesion have never been studied in such a design as ours. Based on earlier studies, we expected that xylitol would interfere with polysaccharide-mediated cell adherence^(24,47)^, but we found no significant differences in adhesion at studied xylitol concentration. Also, adhesion to parotid saliva-coated HA was unaffected by xylitol.

*S. mutans* forms a small portion of the developing oral microbiota – if it is present at all^(48)^ – and expanding our studies to other oral pathogens and commensals could provide a broader understanding of the effects of HMOs and GOS. In addition, biofilm formation could have been evaluated to increase understanding beyond initial stages of colonisation. Human breast milk increases *S. mutans* biofilm formation, whereas individual milk components have various effects, with casein increasing and lactoferrin and IgA (the latter at high concentrations) decreasing biofilm formation and lactose having no effect on it^(20)^. However, although the proportion of mutans streptococci in the oral microbiota may be small, they are virulent cariogenic bacteria and key factors in dental caries^(12)^, underscoring the importance of our findings. Xylitol was used as a reference substance, because it is a prebiotic^(49)^ and because its effects on reducing growth and adherence of mutans streptococci are well documented^(18)^; and lactose, because it is the major carbohydrate component in breast milk and infant formulas^(50,51)^. Growth was assessed continuously, and adherence to parotid saliva-coated HA surfaces and exopolysaccharide-mediated adhesion to a glass surface was evaluated.

Within the limitations of the present study, we showed that 2′-FL and GOS strain-dependently decrease *S. mutans* adhesion or have no effects on it. 2′-FL was not utilised by the *S. mutans* strains that we tested, whereas they grew well with GOS and lactose. Delaying the early colonisation of *S. mutans* decreases the future risk of caries in children^(15,16)^. Thus, 2′-FL, as a compound that is present at high concentrations in human breast milk, may be beneficial for the oral health of infants by not promoting *S. mutans* adhesion. Also, being non-fermentable by *S. mutans*, 2′-FL may decrease acid production in the dental plaque, if it has already colonised erupting teeth. In the limits of this *in vitro* study, and in the perspective of caries pathogenesis, it can be concluded that the ability of 2′-FL to limit the growth and inhibit the adhesion of *S. mutans* may bring an advantage to use this milk oligosaccharide in infant formulas.
